# Temporal changes in medical student perceptions of their clinical skills and needs using a repeated self-assessment instrument

**DOI:** 10.1186/s12909-021-02985-1

**Published:** 2021-10-29

**Authors:** Patrick Barlow, Robert Humble, Amal Shibli-Rahhal

**Affiliations:** 1 Department of Internal Medicine, University of Iowa Carver College of Medicine, 1216H MERF, 375 Newton Rd, IA 52242-2600 Iowa City, USA; 2 Department of Pathology, University of Iowa, IA Iowa, USA

**Keywords:** Clinical clerkship, Self-assessment, Clinical training, Clinical curriculum

## Abstract

**Background:**

Medical student needs in clinical skill training may change over time, but data on this topic are limited. This study uses repeated self-assessments on clinical rotations during medical school to evaluate students’ perceptions of their clinical skill growth.

**Methods:**

A self-assessment rating was completed by students during each clinical rotation as they progressed through their core clinical rotation year. The instrument consisted of questions on 5 clinical skill categories where students rated their performance as “below”, “at” or “above” expected, and open-ended questions on strengths and challenges. We evaluated changes in self-ratings between the first (n=136) and third (n=118) quarters by matched-pair analysis of the shift in responses between time points using a Sign Test. We also identified the main themes from the students’ responses to open-ended questions.

**Results:**

We found 22.4 % and 13.3 % of students increased their self-assessment ratings on “Oral Presentation Skills” and on “Differential Diagnosis”, respectively. In contrast, perceived ability to communicate with patients saw the largest negative shifts. “Patient Interaction” was the most commonly identified area of strength and “Knowledge and Organization” was most frequently cited as a barrier.

**Conclusions:**

Students demonstrated a positive shift in perceived competence in some core clinical skills that are not strongly emphasized in the preclinical curriculum, likely reflecting increased exposure over time. However, their perceived competence in communication skills declined. This may reflect initial over-estimation or true decline due to competing needs/interests. These patterns of change can inform the design of longitudinal curricula that anticipate and address students’ needs during clinical rotations, such as placing increased emphasis on presentation skills and differential diagnosis earlier in the curriculum, and adding more emphasis to communication skills in later phases.

**Supplementary Information:**

The online version contains supplementary material available at 10.1186/s12909-021-02985-1.

## Background

Learner self-assessment is an important component of the formative evaluation necessary to the development of a student’s lifelong learning skills [1]. Lifelong learning is particularly important in the medical profession given that physicians have a professional obligation to maintain adequate knowledge and skills, which often relies on their willingness and ability to engage with self-directed learning [2]. This, combined with the rapid changes and advances in the field of medicine and health sciences, makes the ability to engage in self-directed learning a necessary skill. While opinions on the accuracy of individual self-assessments are mixed [3], the general ability to self-assess is widely considered an important learning tool for developing physicians [4]. In fact, self-assessment is associated with enhanced learner motivation and self-regulated learning [5–10]. Self-efficacy, a different but related concept that refers to an individual’s confidence in their ability to demonstrate a certain performance [11], has also been shown to play an integral role in learners’ motivation and engagement with the learning process [12–15]. Consequently, an argument could be made for medical schools and educators to adapt some of their longitudinal curricula to the evolving self-perceived needs of students, in order to promote optimal engagement and learning. However, studies on the topic of self-assessment in clinical education tend to be specialty-specific and limited to within individual clinical rotations. Additionally, most studies on self-assessment have collected student data at a single timepoint without follow-up [16], so how self-assessment changes as students’ progress through their clinical rotations has not been evaluated.

The present study aims to explore the potential to measure student perceived clinical growth over the course of their core clinical rotations, regardless of rotation specialty, using self-assessment ratings on a standard mid-rotation assessment instrument.

## Methods

### Setting

The University of Iowa Carver College of Medicine admits roughly 145 students to its Doctor of Medicine program each year. The curriculum consists of three main phases. Phase I is 18 months long and consists of the preclinical curriculum. This is followed by Phase II where students spend twelve months, divided into four 12-week blocks, rotating on nine core clinical rotations: (1) Inpatient Internal Medicine (IM), (2) Pediatrics, (3) General Surgery, (4) Obstetrics & Gynecology (OB/GYN), (5) Psychiatry, (6) Neurology, (7) Outpatient Internal Medicine (OIM), (8) Family Medicine, and (9) Community-Based Primary Care (CBPC). During Phase II, students also participate in 2 short non-core rotations of their choosing. Finally, students move into the 18-months long phase III where they participate in advanced (subinternship, intensive care, emergency medicine) and elective clinical rotations and courses.

### Instrument development

The mid-rotation assessment instrument was created in close consultation with core clinical rotation directors, medical students, curriculum leadership, and faculty with program evaluation and assessment expertise. We started by conducting a survey of the stakeholders to assess the most crucial elements that should be addressed in the mid-rotation assessment, and those responses were discussed during several meetings with the different stakeholders until consensus was achieved. The instrument was ultimately designed to gather three pieces of information. The first part of the instrument is a student self-assessment of their perceived performance in five categories, with several items under each category: (1) Knowledge/Clinical Reasoning (3 items), (2) Clinical Evaluation Skills (3 items), (3) Data Presentation Skills (2 items), (4) Studying Skills (3 items), and (5) Team Work (3 items). Students provide an ordinal rating (1 “Below expected level”, 2 “At expected level”, 3 “Above expected level”) of their perceived abilities across these areas as well as written comments on their perceived strengths, weaknesses, and plans for improving. We did not provide a definition of “expected level” to the students as we were interested in capturing their perception of their performance as they self-define it. The second part of the instrument evaluates the student’s progress towards meeting the rotation requirements such as their required clinical experiences. The third part is a narrative (i.e., written) feedback provided by the clinical preceptor or rotation director on strengths and strategies for improvement and continued development that is informed by the student self-assessment. While not the only source for feedback, the self- assessment component of the instrument aims to guide this narrative feedback and make it learner-centered. The specific questions on the instrument were piloted with a small group of students and faculty evaluators. After 3 months of use, the student-self-assessments and narrative clinical preceptor feedback were analyzed and shared with the stakeholders. Additional training to evaluators was provided based on these analyses.

### Data collection

The mid-rotation feedback process constitutes a required element for medical school accreditation in the United States. Consequently, during each of the nine core clinical rotations of Phase II, the students participate in a mandatory mid-rotation feedback session with their primary clinical preceptor or rotation director. Students complete the self-assessment portion of the instrument shortly before these meetings and submit the form electronically to a central tracking application. Thus, each student completes a total of nine self-assessments over the course of 12 months. The present study focuses on the student self-assessment portions of the mid-rotation feedback instrument (Additional file 1) using data stored in the central tracking application. The self-assessment forms were de-identified by a curriculum manager and each student was assigned a random number that was placed on their forms to allow data matching. The researchers involved in this study had no access to student information. This study was reviewed by the University of Iowa Institutional Review Board (IRB) and was determined not to meet the federal regulatory definition of human subjects research.

### Data analysis

We compared student self-assessments performed during the first 12-week block of Phase II to those performed during the third block. We pursued an embedded mixed-methods design in which the analysis of the self-assessment data, which included a quantitative analysis of the matched and unmatched ratings, were then bolstered by a comparative qualitative content analysis of open-ended comments [17].

#### Self-assessment ratings

Self-assessment ratings were analyzed with a combination of descriptive statistics including frequencies and percentages. The percent change from the first to third 12-week block (Block 1 and Block 3 respectively) was assessed for the whole sample by category and by individual items within each category. A matched-pair analysis of the *shift* in responses between time points was analyzed using a Sign Test, which evaluates each matched pair of ratings for negative differences, positive differences, and ties. An example of each in the context of our study is found in the table below. These matched pairs were defined as a single student’s Block 1 and Block 3 scores, and all students for whom we could *not* match a Block 1 and Block 3 score were excluded from that particular analysis. As there are no readily available measures of effect size for many non-parametric tests including the Sign Test, we used the procedures described by Rosenthal and Rubin [18] to calculate a *r*_equivalent_ value for any statistically significant result. They described their procedure was acceptable in cases where, “…(a) the alternative is to have no effect size estimate at all (e.g., only sample sizes and p values are known for a study), [and] (b) nonparametric procedures were used for which there are no currently accepted effect size indicators (p. 496)”. The values are interpreted as a point-biserial correlation between each pair of variables where values of |0.1|, |0.3|, and |0.5| would be considered small, medium, and large effects, respectively, using the conventions laid out by Cohen [19]. Statistical tests were 2-sided and *p* < 0.05 was considered statistically significant. Analysis was completed using SPSS v25 (IBM, Inc.).

**Table:** 

Negative Difference	3 “Above expected level”	2 “At expected level”
Positive Difference	1 “Below expected level”	2 “At expected level”
Tie	2 “At expected level”	2 “At expected level”
Sign Test Outcome	First Rating	Second Rating

#### Open-ended comments

Qualitative content analysis is a flexible method for analyzing text data that varies in approach depending on the specific problem being studied [20]. Conventional content analysis involves deriving salient themes/codes directly from the text while directed content analysis relies on previous data and theory to develop themes/codes a priori and apply them to the text [20]. We use both approaches in this study. Conventional content analysis was used with Block 1 data because we did not make assumptions about the types of strengths and barriers that the students would share. We then used the codes developed from the first set of data and applied them to a directed content analysis of Block 3 data, so that direct comparisons could be made between the two time points.

All data were entered into Microsoft Excel for analysis. Then, both sets of data were analyzed independently by two raters (RH, PB). Disagreements were resolved to obtain a final consensus coding. The frequencies of each code were compared across the two time points to observe the progression of students’ perceived skills as they gained more experience in their core clinical year. The prevalence of each code was also compared across each of the different rotations. Results are summarized using frequencies and percentages as well as representative comments unique to each theme.

## Results

Since completion of the mid-rotation assessment instrument was a required part of each rotation, our samples for Block 1 (*n*= 136) and Block 3 (*n*= 118) reflect 100 % of the students taking the applicable rotations during the time the forms were collected. The discrepancy between the numbers of students in Block 1 and 3 is due to some students being on a leave of absence from their studies and to others participating in short non-core rotations during Phase II of the curriculum where mid-rotation feedback is not required. Of these 254 total forms, 100 were successfully matched between a student’s Block 1 and Block 3 self-assessment ratings. Unmatched students were those who did not participate in any clinical rotations or were scheduled to take a non-core rotation that did not require the self-assessment instrument to be completed during one of the collection periods. Table 1 shows the breakdown of each sample by rotation.

**Table 1:** Proportion of Students in Each Clinical Rotation by Sample

Rotation	Frequency (%) by Sample		
	**Block 1 (** ***n*** **= 136)**	**Block 3 (** ***n*** **= 118)**	**Total (** ***N*** **= 254)**
Community-Based Primary Care (CBPC)	12 (8.8 %)	10 (8.5 %)	22 (8.7 %)
Family Medicine	11 (8.1 %)	14 (11.9 %)	25 (9.8 %)
Inpatient Internal Medicine (IM)	18 (13.2 %)	15 (12.7 %)	33 (13.0 %)
Neurology	15 (11.0 %)	9 (7.6 %)	24 (9.4 %)
Obstetrics & Gynecology (OB/GYN)	19 (14.0 %)	16 (13.6 %)	35 (13.8 %)
Outpatient Internal Medicine (OIM)	11 (8.1 %)	13 (11.0 %)	24 (9.4 %)
Pediatrics	21 (15.4 %)	17 (14.4 %)	38 (15.0 %)
Psychiatry	15 (11.0 %)	9 (7.6 %)	24 (9.4 %)
Surgery	20 (14.7 %)	15 (12.7 %)	35 (13.8 %)

### Self-assessment ratings

**Fig. 1:**
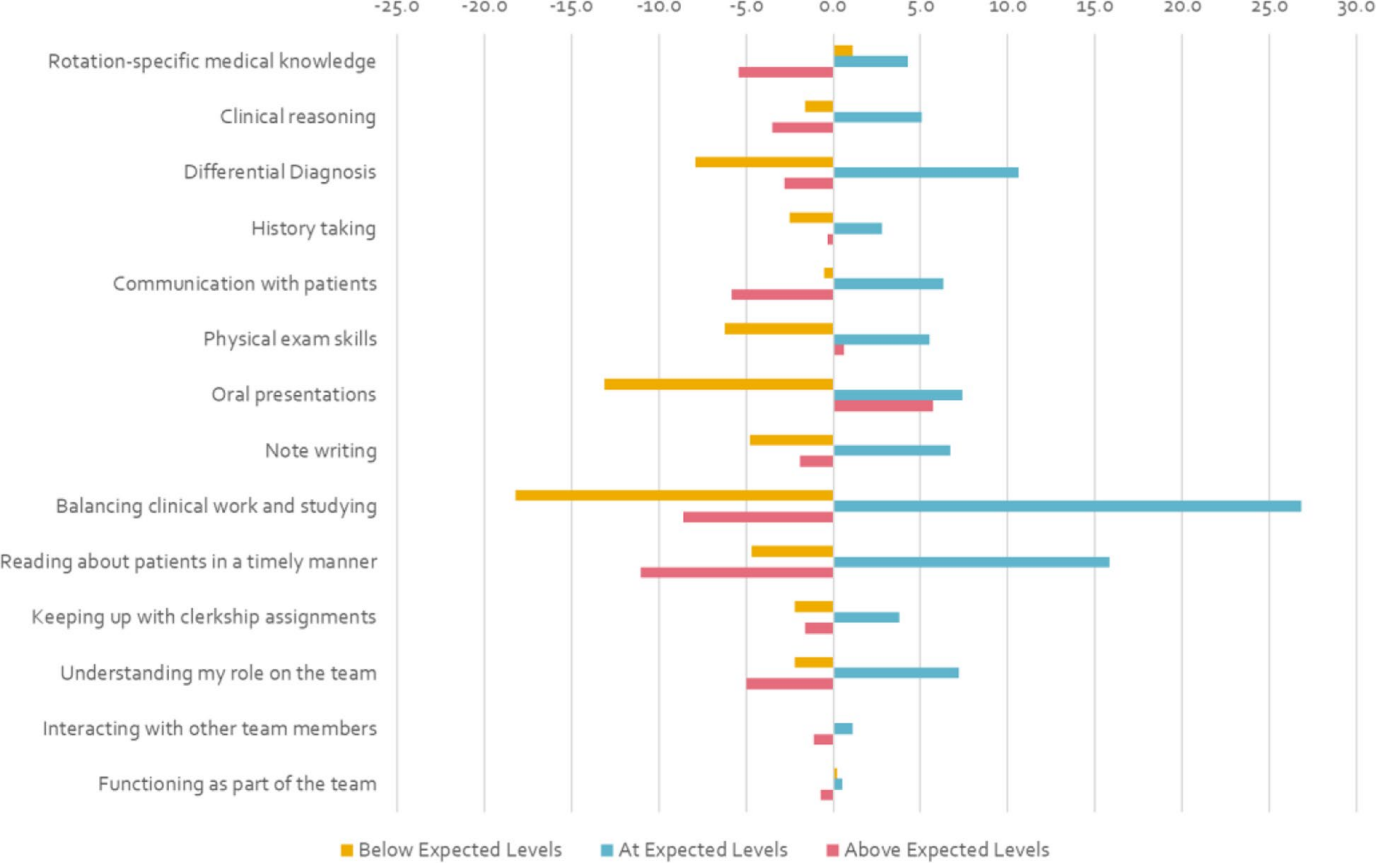
Percent-Changes in Student Self-Assessment Ratings between First and Third 12-Week Block by Item (Unmatched)

Figure 1 displays the changes in frequency for each of the self-assessment ratings in the unmatched (i.e., full) sample. Within each item, we observed an increase in the proportion of students rating themselves “At expected level” from Block 1 to Block 3. This was paralleled with a decrease in the proportion of students rating themselves “Below expected level” on several items, specifically “Differential Diagnosis”, “Oral Presentations”, and “Balancing Clinical Work and Studying”. On the other hand, we also saw a decreased in the proportion of students rating themselves “Above expected level” for every item except “Physical Exam Skills” and “Oral Presentations”.

**Fig. 2:**
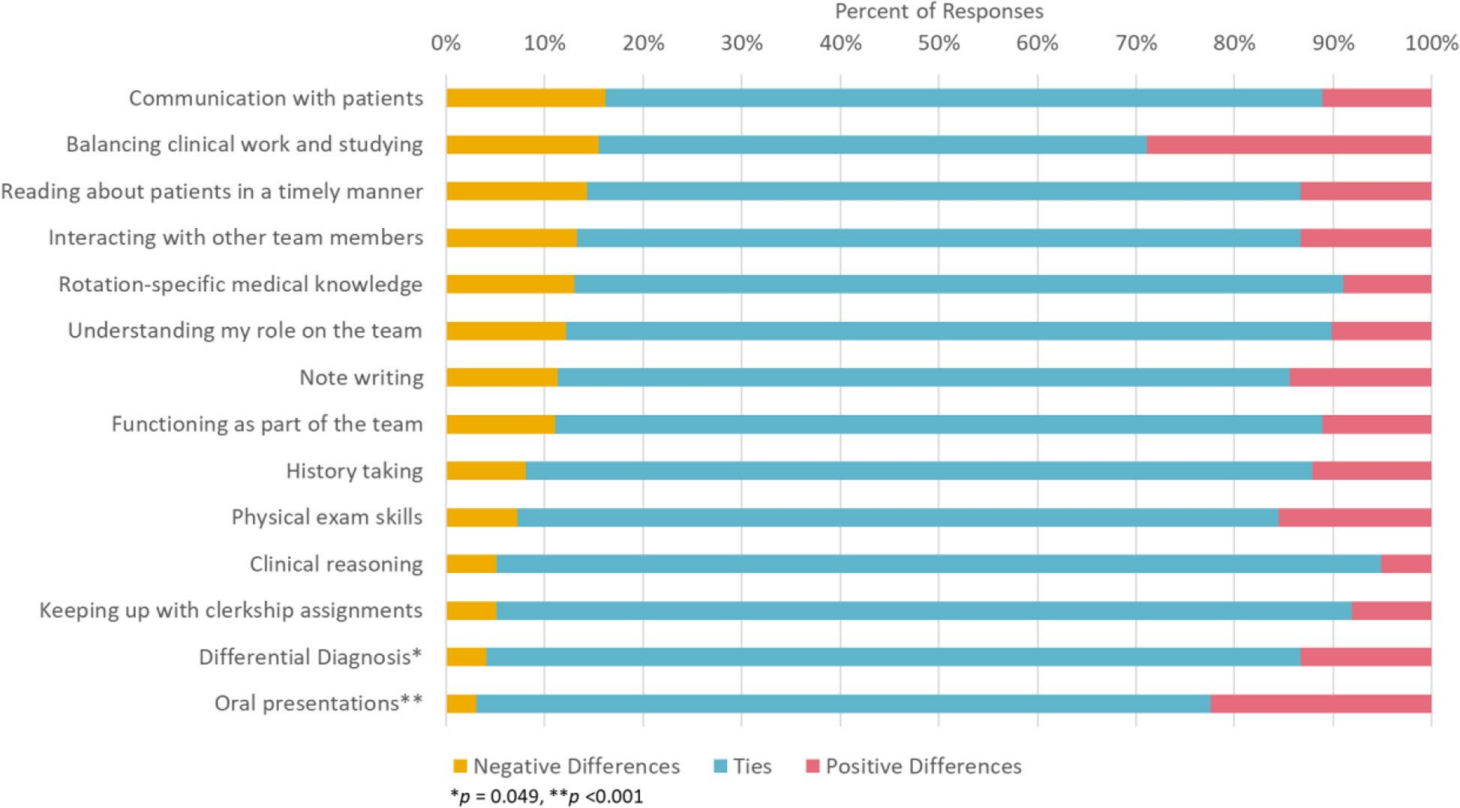
Differences in Student Self-Assessment Ratings between First and Third 12-Week Block (Matched)

The matched comparisons of 100 students’ Block 1 and Block 3 ratings yielded results that largely supported the percent-change results from the unmatched sample (Fig. 2). We found a statistically significant number of *positive* shifts in self-assessment ratings on “Oral Presentation Skills” from Block 1 to Block 3, with 22.4 % of students increasing their rating by at least one level (*r*_equivalent_ = 0.36, *p* < 0.001). We also saw a significant positive shift in self-assessment ratings on “Differential Diagnosis”, with 13.3 % of students increasing their rating on this skill (*r*_equivalent_ = 0.17, *p* = 0.049). In contrast, students’ perceived ability to communicate with patients saw the largest proportion of *negative* shifts from Block 1 to Block 3. While statistically significant, it is important to note that the effect size estimates, for “Differential Diagnosis” in particular, are relatively small.

### Reflective comments

Seven primary themes across the three open-ended items were identified in the initial sample (Block 1) and then applied to the second sample (Block 3) through directed content analysis (Table 2). Patient interaction was the most commonly identified area of strength/confidence in both samples with 37.2 % and 43.9 % of coded segments falling into this theme in Block 1 and Block 3, respectively. Statements such as, *“I feel confident in history taking and providing an outlet for the patient to speak because I tend to use open-ended questions and convey empathy quite easily”* (Student 33, Block 1), were a common expression of this theme both in Block 1 and Block 3. The theme was the most prevalent one in every rotation, but it appeared the most in the OB/GYN and Pediatrics rotation comments.

Knowledge and Organization was the theme most frequently cited as a barrier at both time points, but more so in Block 3 (23.6 % versus 28.9 %). Many of the comments in this theme were related to rotation-specific knowledge or the sheer volume of information there was to learn. One such comment was, *“Time management - I feel like there is an incredible breadth of information I need to learn in OB/GYN that it is difficult for me to know how to learn it all while also being in a clinical setting. I also have responsibilities outside of clinical rotations that I am trying to juggle”* (Student 75, Block 1).

**Table 2:** Proportion of Coded Segments Associated with Seven Primary Themes

Theme: *Description*	% of Coded Segments by Quarter			
	**Confidence**		**Barriers**	
	**Block 1** **(** ***n*** **= 288)**	**Block 3** **(** ***n*** **= 205)**	**Block 1** **(** ***n*** **= 178)**	**Block 3** **(** ***n*** **= 114)**
**Patient interaction**: *Refers to interpersonal communication skills and interviewing skills required with patient care such as taking a pertinent history and building rapport.*	37.2	43.9	11.8	3.5
**Documentation/Reporting**: *Refers to ability to document and communicate clinical findings through written (notes) and oral (presentations) formats.*	16.7	17.6	15.2	6.1
**Team/Structure/Workflow**: *Refers to ability to work within the healthcare team (point of confidence) or trouble with personalities, expectations, or systemic issues as barriers.*	17.0	11.7	18.5	17.5
**Knowledge and Organization**: *Refers to medical knowledge broadly as well as rotation-specific knowledge. Also includes study skills and work/life balance associated with studying.*	13.2	7.8	23.6	28.9
**Physical exam**: *Refers to physical exam skills that are required during routine clinical encounters.*	5.6	9.8	11.2	10.5
**Clinical reasoning/information synthesis**: *Refers to ability to interpret clinical information and synthesize it into a differential diagnosis and management plan.*	5.6	6.3	0.0	8.8
**Inexperience or Lack of Practice**: *Refers to expression of lack of confidence in certain skills due to limited experience and/or a need to continue practicing.*	0.0	0.0	8.4	17.5

Additional representative quotes from each theme for points of strengths and barriers are listed in Table 3.

**Table 3:** Results of Content Analysis for Student Reflective Comments for Strengths and Areas of Confidence

Theme	Representative Quote(s) for Confidence/Strength	Representative Quote(s) for Barriers and Areas for Improvement
Patient interaction	*“I feel confident in history taking and providing an outlet for the patient to speak because I tend to use open-ended questions and convey empathy quite easily.”* (B1)	*“Obtaining & organizing chronological information obtained from patients and collateral because I sometimes have trouble following patient stories when given a lot of info or out of order.”*
Documentation/Reporting	*“Oral presentations - I think that I present effectively and with enough detail while still reserving additional details included in the notes.”* (Q?)	*“Presentations: Still unsure what info is more relevant and still learning what normal/abnormal lab values look like.”*
Team/Structure/Workflow	“*Communicating with the healthcare team with questions and patient information because I was told my presentations were clear and concise.”* (B1) *“Communicating with team members especially the residents - asking what I can do to help to clarify expectations for notes, presentations; Speaking up if I don’t understand something or have a question.”* (B3)	*“Not knowing what I should be doing. I usually ask, but at times I don’t want to annoy others/make more work for them. (Also I do know I’m paying a lot of money to be here so that usually helps me to remind myself of this.)”* (B1) *“[Electronic Medical record] being confusing, repeated practice, residents and staff [not] communicating expectations clearly”* (B3)
Knowledge and Organization	*“I believe I’m doing well in my studying, having made time to read through the case files book and complete a substantial number of [practice] questions so far.”* (Q?)	*“Time management - I feel like there is an incredible breadth of information I need to learn in OB/GYN that it is difficult for me to know how to learn it all while also being in a clinical setting. I also have responsibilities outside of clinical rotations that I am trying to juggle.”*
Physical exam	*“I feel confident in my general pertinent physical exam skills and am getting more confident in the pelvic exam. This is through good feedback on what I am doing well/how I can improve.”* (B1) *“Physical exams: I know the exams and I am beginning to see some abnormal physical exams which is helping.”* (B3)	*“Time/experience: some practice with Simulated Patients is not enough to feel confident in the physical exam skills we learned or knowing how to distinguish between similar patient presentations.”* (B1) *“I need to become more proficient with physical examination maneuvers associated with orthopedic complaints.”* (B3)
Clinical reasoning/information synthesis	*“I feel confident in initially evaluating a problem list and setting a logical agenda to follow through the history taking process.”* (B1) *“Formulating differential diagnoses with pertinent history and physical information by developing clinical evaluation skills in general medicine.”* (B3)	*“Broader knowledge base of medicine - forgot from pre-clinical; difficulty managing more complicated patients.”* (B1)
Confidence/anxiety/experience/practice	*N/A*	*“Shyness in requesting feedback for fear of harsh criticism; Drawing a blank when asked for an assessment and plan.”* (B1)

## Discussion

This is the first study examining changes in students’ self-assessment longitudinally across multiple rotations over the course of their core clinical rotations. While the self-assessment component of our instrument was primarily aimed at guiding the narrative feedback and keeping it learner-centered, our review of the students’ responses revealed interesting trends over time. Students demonstrated a significant positive shift in their perceived competence in core clinical skills such as developing differential diagnoses and delivering oral presentations. These shifts in ratings were partially supported by a significant decline from 17.6 % to just 6.1 % of students highlighting Documentation/Reporting skills as a barrier in their reflective comments. These improvements are not surprising given that practicing oral presentations and developing differential diagnoses are essential components of the core clinical rotations [21]. One study found that clinical faculty rated the expected baseline skill for oral presentations and differential diagnosis for students starting their rotations as lower than both their preclinical faculty colleagues and students themselves, which suggests an acknowledgment that these clinical skills are meant to be developed over the core year [22].

On the other hand, there was a significant negative shift in the students’ perceived competence in their provider-patient communication skills. In fact, 16.2 % of students rated themselves lower on these skills at Block 3 compared to Block 1, nearly all from “Above expected level” to “At expected level”. One potential explanation for this drop, is that students may have started by over-rating their competence in this domain then moderated their perceived skills over time as they gained more experience. Such behavior would somewhat be expected given that communication skill training is highly emphasized during the preclinical phase of the curriculum, but relies heavily on scripted clinical encounters with simulated patients. In this setting, it is not surprising that students may highly rate their skills at the beginning of the clinical phase, only to be later challenged by actual clinical encounters. This explanation is also supported by previous studies that concluded students had a tendency to overestimate their skills in certain areas when self-assessing [3]. Another possible explanation may be that the observed change in ratings represents a true decline in communication skills as a result of negative role-modeling, time constraints, and competing interests once students start spending more time in actual clinical settings [23].

While it may not be possible to fully explain or rationalize the shifts is students’ self-ratings over time, it is important to keep in mind that these ratings still reflect the students’ perceptions of their performance and needs at different time points. As a matter of fact, we acknowledge that these individual perceptions may be anchored in a myriad measurable and unmeasurable factors including-but not limited to- self-defined performance targets, self-comparison to peers, and prior feedback received from patients or preceptors. Focus-group to identify from the students the range of inputs which caused changes in scores over time could have enhanced our understanding of the observed trends, and may be performed at a later stage as we continue to evaluate ways to modify the curriculum to meet student needs. Such analyses were not conducted for the purpose of this study as the focus here is on identifying student perceptions rather than explaining them.

We actually chose not to provide students with instructions on what defines “expected level” on the assessment instrument in order to collect a more accurate individual “needs assessment”, in an effort to introduce student perceptions into the assessment of student performance, and to have it guide a learner-centered feedback process.

Medical training relies heavily on educator assessment as the primary mode to guide curriculum design and determine student success and achievement of clinical competency, with less value attached to learner self-assessment. In fact, the correlation between learner self-assessment and educator assessment of clinical skills is quite variable, with some studies showing some degree of correlation [24–27] and others showing very weak or no correlation [28–30]. While it is tempting to use these data to dismiss student self-assessment and perceptions as inaccurate, it is important to note that self-efficacy has been shown to be at least as important as the presence of knowledge and skill to student academic motivation [14, 31, 32]. Consequently, educators would do well to tailor clinical curricula to meet the learners’ perceived needs while still fostering acquisition of competency. For example, the findings from our study that we discuss in the previous paragraphs may be used to re-design clinical or even pre-clinical curricula in ways that place increased emphasis on presentation skills in the earlier phases and on communication skills in later phases.

Care should be exerted when generalizing these findings however, since our study was limited by its observational nature, by the fact that it took place at a single institution and by the rather limited sample size. Additionally, we used self-assessment ratings as a proxy for actual ability. Finally, we had no control over the order in which students took their rotations over the study period, so we cannot expect all students to have had the same experiences between the two measurement points. We worked to mitigate these limitations by using a repeated-measures design that compares students’ Block 1 rating to their own Block 3 rating, by employing multiple data sources to triangulate our findings, and by sampling across the whole student cohort rather than focusing on a single specialty’s rotation. Furthermore, all core clinical rotations follow a similar structure and they all offer comparable student involvement in care, educational activities, and grading criteria; therefore, the core progression in training should be equivalent across our sample despite having different rotations during Block 2.

## Conclusions

In summary, student self-perceived needs and challenges during clinical rotations evolve over time; and the findings from this study may be used to inform the design of clinical curricula. While individual clinical rotations aim to introduce students to knowledge and practices specific to certain clinical disciplines (such as surgery, pediatrics etc.), it may be possible to create and reinforce a more unified clinical skills curriculum across different rotations. For example, one could envision a parallel curriculum that strongly emphasizes clinical reasoning and note writing early in the clinical phase, regardless of what rotations the students are on, and that revisits communication skills at a later stage in order to address the real-life challenges that students would have experienced by then.

## Supplementary Information

**Additional file 1.**

## Data Availability

The datasets generated during and/or analyzed during the current study are not publicly available student confidentiality.
