# Supplementary material for: Temporal changes in medical student perceptions of their clinical skills and needs using a repeated self-assessment instrument
Source: BMC Med Educ. 2021 Oct 29;21:550. doi: 10.1186/s12909-021-02985-1 (PMC8555323; doi:10.1186/s12909-021-02985-1)
Supplement: Supplementary file 1 — Additional file 1. [file 12909_2021_2985_MOESM1_ESM.docx]

Additional file 1

**Mid-Rotation Student Self-Assessment**

***Rotation name***

Complete this self-assessment prior to your mid-rotation feedback meeting

| Student name: |  | Date: |  |
| --- | --- | --- | --- |

| *For each of the following domains, I am performing:* | | | |
| --- | --- | --- | --- |
| **Domain** | **Below expected level** | **At expected level** | **Above expected level** |
| 1. **Knowledge/Clinical Reasoning** | | | |
| 1. Rotation-specific medical knowledge |  |  |  |
| 1. Clinical reasoning |  |  |  |
| 1. Differential diagnosis |  |  |  |
| 1. **Clinical evaluation skills** | | | |
| 1. History taking |  |  |  |
| 1. Communication with patients |  |  |  |
| 1. Physical exam skills |  |  |  |
| 1. **Data presentation skills** | | | |
| 1. Oral presentations |  |  |  |
| 1. Note writing |  |  |  |
| 1. **Studying skills** | | | |
| 1. Balancing clinical work and studying |  |  |  |
| 1. Reading about patients in a timely manner |  |  |  |
| 1. Keeping up with rotation assignments |  |  |  |
| 1. **Team work** | | | |
| 1. Understanding my role on the team |  |  |  |
| 1. Interacting with other team members |  |  |  |
| 1. Functioning as part of the team |  |  |  |

| **I am on target to meet end-of-rotation RCEs** | Agree | Disagree | Not sure |
| --- | --- | --- | --- |

| **Behaviors/skills I feel confident in and why** *(list up to 3)* |
| --- |
|  |
| **Areas for improvement related to my performance/experience** |
| Barriers/issues impeding my performance: |
| Areas that need improvement and my plan to improve them: |
